# Health Literacy and Patient-Reported Outcomes Measurement Information System Scores Among Patients Referred to Spine Surgeons

**DOI:** 10.7759/cureus.80717

**Published:** 2025-03-17

**Authors:** Colin E Webster, Elizabeth Stiles, Aurora Scotti, Yong H Kim, Charla R Fischer

**Affiliations:** 1 Orthopedic Surgery, New York University (NYU) Grossman School of Medicine, New York, USA

**Keywords:** adi, area deprivation index, health literacy, mychart, orthopedic spine surgery, orthopedic surgery, promis, spine, spine surgery

## Abstract

Background

Health literacy is “the degree to which individuals can obtain, process, and understand basic health information and services to make appropriate health decisions." Low health literacy is associated with adverse health outcomes, such as increased risk and length of hospitalization after abdominal surgery. However, the impact of health literacy on outcomes in the spine surgery patient population is understudied. This study seeks to evaluate the relationship between patients' health literacy scores and various outcomes, primarily a patient's Patient-Reported Outcomes Measurement Information System (PROMIS) score at their baseline visit with a spine surgeon. A greater understanding of the impact of health literacy on health outcomes may improve treatment for patients with lower health literacy.

Methods

This is a single-center retrospective study at New York University (NYU) Langone Orthopedic Center. A health literacy measurement survey (i.e., the Newest Vital Sign survey) was administered to English-speaking adult patients aged 18 years and older who presented to two attending spine surgeons between June 1, 2022, and August 15, 2022, as new or follow-up patients. The survey consists of six questions, and patients were categorized into two different health literacy levels based on the number of correct responses. A score of 0-3 suggests limited literacy, and a score of 4-6 indicates adequate literacy. Additional data collected include PROMIS at the patient’s baseline appointment with the surgeon to create consistency between new and follow-up patients. Key demographic and clinical data were also collected. Univariate associations between health literacy and PROMIS scores were investigated using the Welch Two Sample t-test and Pearson's Chi-squared test. A multivariate analysis was carried out implementing a binary logistic regression model.

Results

This study included 57 patients with an average age of 57 years, 29 (51%) of whom identified as female. The racial breakdown of this cohort was 33 (58%) White, 11 (19%) Black, 5 (9%) Asian, and 5 (9%) Hispanic. The health literacy survey results demonstrated that 25 (44%) had limited health literacy, and 32 (56%) had adequate health literacy. Limited literacy patients were older (mean age of 62 years for Limited vs. 54 years for Adequate, P=0.024) and more likely to be patients of color (either Asian, Black, or Hispanic) (15 (60%) Limited vs. 6 (19%) Adequate, P = 0.002). Limited literacy patients also, on average, had a lower self-reported physical health score (36.6 for Limited vs. 41.2 for Adequate, P=0.050) and were more likely to have hypertension (20 (80%) Limited vs. 10 (31%) Adequate, P<0.001). A logistic regression model yielded an odds ratio of 1.16 between patient-reported physical health and health literacy, indicating that the odds of having adequate health literacy increase by about 16% for each unit increase in the Physical Health score. A Variance Inflation Factor (VIF) test was used and demonstrated minimal multicollinearity among the variables in the logistic regression.

Conclusion

This study shows that health literacy plays a significant role in health outcomes, especially in chronic health conditions like physical health for spine surgery patients and hypertension. These results align with the literature, showing how lower health literacy correlates with worse physical health scores and a greater incidence of hypertension.

## Introduction

A significant yet currently understudied component of quality medical care is patients’ health literacy. Health literacy is “the degree to which individuals can obtain, process, and understand basic health information and services to make appropriate health decisions” [1]. The 2003 National Assessment of Adult Literacy estimated that 14% of American adults had below basic literacy and an additional 22% had only basic literacy, with a disproportionate amount of those with lower literacy being Black, Hispanic, American Indian/Alaska Native, and Multiracial [2]. Low health literacy is associated with several adverse health outcomes, including increased risk and length of hospitalization, greater reliance on emergency care after abdominal surgery, higher mortality in chronic hemodialysis patients, greater likelihood of minor complications after a radical cystectomy, and worse physical function and mental health [3-9].

Health literacy among surgical patients, especially orthopedic spine surgery patients, is currently understudied [10]. Better identification of health literacy needs among patients referred to spine surgeons could lead to improved patient education efforts that mitigate the adverse health outcomes associated with low health literacy.

A systematic review of the main tools used to evaluate health literacy identified the Newest Vital Sign (NVS) survey as the most frequently used instrument [10]. The NVS is a three-minute, six-question assessment in which patients answer questions about a nutritional label. The power of the NVS lies in the fact that it is validated, highly sensitive, short enough for routine use, and available in both English and Spanish [11].

This study seeks to build on the literature in the health literacy space by evaluating the relationship between patients' health literacy scores and various outcomes, primarily a patient's Patient-Reported Outcomes Measurement Information System (PROMIS) score at the baseline visit with a spine surgeon. The authors hypothesize that lower health literacy will be associated with poorer PROMIS scores and a higher prevalence of chronic health issues like hypertension in patients seeing spine surgeons.

## Materials and methods

Patient population

This is a single-center retrospective study at NYU Langone Orthopedic Center. A health literacy measurement survey (i.e., the Newest Vital Sign survey) was administered to English-speaking adult patients aged 18 years and older who presented to two attending spine surgeons between June 1, 2022, and August 15, 2022, as new or follow-up patients for elective spine surgery. Patients with any physical or psychological condition that would impair active participation in the study were excluded, according to the treating clinician. Institutional review board approval was obtained prior to beginning the study.

Data collection

**Figure 1:**
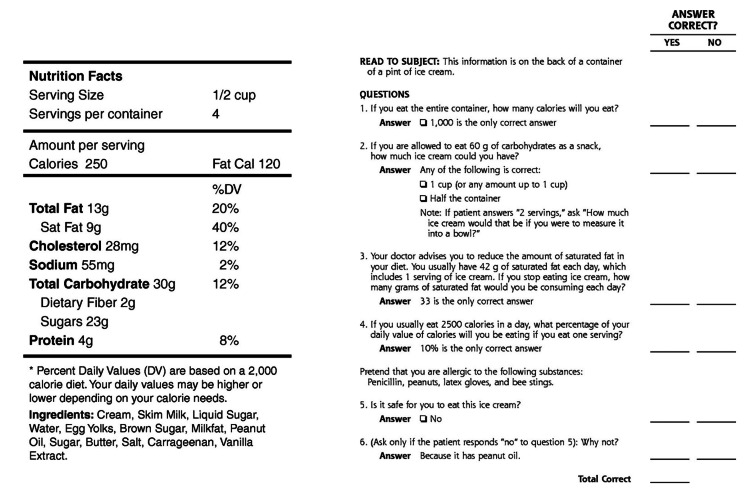
Newest vital sign survey and nutrition label.

The NVS survey consists of six questions based on information captured on a nutrition label (Figure 1). Study participants were categorized into two different health literacy levels based on the number of correct responses to the survey. A score of 0-3 suggests limited literacy, and a score of 4-6 almost always indicates adequate literacy [11].

The patient electronic medical record system from our institution (Epic Caboodle, Version 15; Verona, Wisconsin) was utilized to collect data regarding patient demographic and clinical variables.

Patient demographic data collected includes age at the time of appointment, sex, race, state area deprivation index (ADI) (scored between 1 and 10, with 10 indicating a more deprived area), national ADI (scored between 1 and 100, with 100 indicating a more deprived area), and MyChart access and usage. MyChart access was defined as the patient having positive read receipts in their chart, and usage was defined as the patient having asked a question via MyChart to a provider.

Patient clinical data collected includes multiple PROMIS scores at the patient’s baseline appointment with the surgeon, including Global Mental Health, Global Physical Health, Anxiety, Depression, Physical Function, Pain Intensity, and Pain Interference. For these scores, better health outcomes are associated with higher scores for Global Mental Health, Global Physical Health, and Physical Function, and with lower scores for Pain Intensity, Pain Interference, Anxiety, and Depression. Additional clinical data includes hypertension, chronic kidney disease (CKD), diabetes, and whether or not the patient was a current smoker.

## Results

A total of 57 patients were included in this study with an average age of 57 years and 29 (50.9%) identifying as female. The racial breakdown of this cohort was 33 (61.1%) White, 11 (20.4%) Black, 5 (9.3%) Asian, and 5 (9.3%) Hispanic. The health literacy survey results show that 25 (44%) had limited health literacy and 32 (56%) had adequate health literacy. Limited literacy patients were older (mean age 61.92 years for Limited vs. 53.50 years for Adequate, P=0.024) and more likely to be patients of color (Asian, Black, or Hispanic) (15 (60%) Limited vs. 6 (18.75%) Adequate, P = 0.002). Limited literacy patients also, on average, had a lower self-reported PROMIS global physical health score (36.57 for Limited vs. 41.26 for Adequate, P=0.050) and were more likely to have hypertension (20 (80%) Limited vs. 10 (31.2%) Adequate, P<0.001) (Table 1).

**Table 1:** Comparison of limited literacy and adequate literacy. PROMIS: Patient-Reported Outcomes Measurement Information System; ADI: Area deprivation index.

Variable	Overall - Mean (SD)	Limited Literacy - Mean (SD)	Adequate Literacy - Mean (SD)	P-value
n	57	25	32	
Age (mean (SD))	57.19 (14.25)	61.92 (12.86)	53.50 (14.38)	0.024
Sex (%)				
Female	29 (50.9)	13 (52.0)	16 (50.0)	1
Male	28 (49.1)	12 (48.0)	16 (50.0)	
Race (%)				
Asian	5 (9.3)	4 (17.4)	1 (3.2)	0.002
Black	11 (20.4)	6 (26.1)	5 (16.1)	
Hispanic	5 (9.3)	5 (21.7)	0 (0.0)	
White	33 (61.1)	8 (34.8)	25 (80.6)	
State ADI	3.22 (2.33)	3.54 (2.72)	2.97 (1.99)	0.39
National ADI	15.85 (20.30)	19.04 (25.30)	13.39 (15.38)	0.341
PROMIS 10 Global v1.2 Mental Health	46.82 (10.95)	43.84 (11.75)	49.21 (9.92)	0.155
PROMIS 10 Global v1.2 Physical Health	39.29 (7.50)	36.57 (6.51)	41.26 (7.68)	0.05
PROMIS Anxiety	51.81 (10.85)	50.47 (13.99)	52.73 (8.33)	0.58
PROMIS Depression	50.85 (10.30)	51.71 (12.96)	50.25 (8.28)	0.713
PROMIS Physical Function	37.33 (7.79)	35.06 (6.74)	38.90 (8.20)	0.097
PROMIS Pain Intensity	55.81 (6.73)	56.81 (6.82)	55.10 (6.72)	0.432
PROMIS Pain Interference	65.31 (6.57)	67.68 (5.89)	63.82 (6.63)	0.051
Hypertension (%)				
No	27 (47.4)	5 (20.0)	22 (68.8)	0.001
Yes	30 (52.6)	20 (80.0)	10 (31.2)	
CKD (%)				
No	53 (93.0)	22 (88.0)	31 (96.9)	0.436
Yes	4 (7.0)	3 (12.0)	1 (3.1)	
Diabetes (%)				
No	51 (89.5)	22 (88.0)	29 (90.6)	1
Yes	6 (10.5)	3 (12.0)	3 (9.4)	
Current smoker (%)				
No	52 (91.2)	22 (88.0)	30 (93.8)	0.772
Yes	5 (8.8)	3 (12.0)	2 (6.2)	
MyChart access (%)				
No	13 (22.8)	6 (24.0)	7 (21.9)	1
Yes	44 (77.2)	19 (76.0)	25 (78.1)	
MyChart usage (%)				
No	20 (35.1)	10 (40.0)	10 (31.2)	0.684
Yes	37 (64.9)	15 (60.0)	22 (68.8)	

For the multivariate binary logistic regression model, variables were selected based on whether they demonstrated a significant association (p < 0.05) or were approaching significance in the univariate analyses to investigate their combined relationship with health literacy (Table 1). The final model included three PROMIS scores as predictors (Global Physical Health, Pain Interference, and Physical Function). This model yielded an odds ratio of 1.16 between patient-reported PROMIS global physical health and health literacy, indicating that the odds of having adequate health literacy increase by about 16% for each unit increase in the PROMIS Global Physical Health score (Table 2). To test the validity of this model, a popular tool for analyzing multicollinearity called the Variance Inflation Factor (VIF) test was used [12]. High VIF values indicate that a predictor is highly correlated with other predictors, which can cause instability in the regression coefficients. Using a commonly used threshold of 10, this analysis shows all variables in the logistic regression model with values well below 10, indicating the predictors are reasonably independent of each other, which supports the reliability of the estimated coefficients in the model (Table 3).

**Table 2:** Logistic regression model between select PROMIS scores and health literacy. PROMIS: Patient-Reported Outcomes Measurement Information System.

Variable	OR (CI)	P-value
(Intercept)	0.176 (4.63×10^-10^ - 6.67×10^2^)	0.863
PROMIS 10 Global v1.2 Physical Health	1.16 (1.01-1.34)	0.044
PROMIS Physical Function	0.992 (0.828-1.19)	0.932
PROMIS Pain Interference	0.955 (0.770-1.18)	0.674

**Table 3:** Multicollinearity test of factors in the logistic regression model.

Variable	Variance Inflation Factor (VIF)
PROMIS 10 Global v1.2 Physical Health	1.724183
PROMIS Physical Function	3.410179
PROMIS Pain Interference	2.969819

## Discussion

This study examined the relationship between health literacy and key demographic and clinical outcomes. It sought to advance the field not only by evaluating these relationships but also by collecting more health literacy data, which is not routinely collected among patients, especially those who visit spine surgeons.

Examining the primary relationship between health literacy and PROMIS scores, only one score (Global Physical Health) was significantly correlated with health literacy; lower health literacy was associated with lower physical health. This relationship is consistent with literature in the field and may be due to a greater likelihood of having significant health complications or less access to treatments or support for those conditions [3-9]. A larger sample size may have demonstrated significant relationships between health literacy and other PROMIS scores.

Across demographic data trends, older patients relative to younger patients and patients of color relative to white patients had lower health literacy, respectively. This is consistent with the literature [7,13]. Lower health literacy among older populations may be due to less competency with health literacy resources, often accessed via the internet. Lower health literacy among patients of color may be due to significant barriers to equitable access to educational resources in school and community contexts, as highlighted by Bather JR et al., who found that the racial composition of educational and residential social environments was significantly associated with adult health literacy [13].

The lack of a significant relationship between area deprivation index scores and health literacy is surprising, as there is evidence in the field of a significant relationship between more deprived areas and lower health literacy scores [14]. The absence of a significant relationship in this data may be due to the small sample size and the lack of geographic diversity among patients at this single-center study. One might also assume that low MyChart access or usage would correlate significantly with lower health literacy scores. The lack of significance in this relationship in this study may be due to small sample size and/or the need for a more accurate tool to assess MyChart usage, such as a patient-reported survey.

The significance of the association between health literacy and hypertension has been inconsistent in the field [15]. Thus, this analysis adds to the field by underscoring the significance of the relationship between lower health literacy scores and a greater incidence of hypertension.

There were a few limitations with this study. First, the sample size was small and limited the power of the study. Additionally, the patient population included all patients coming in to see spine surgeons, including those that did not end up getting surgery. A study that controls for a more specific patient population, such as patients undergoing the same surgical procedure, could better control for confounding variables and better assess the influence of health literacy across outcomes.

Moving forward, a future study that seeks to better measure how health literacy impacts health outcomes may explore the relationship between the differences in pre and post-op PROMIS scores and health literacy. This would show whether health literacy influences how much better a patient feels after a surgical procedure, which may drive greater investment in health literacy supports.

## Conclusions

This study demonstrates that health literacy plays a significant role in health outcomes, especially chronic health outcomes like physical health and hypertension, for patients who see spine surgeons. Since chronic health outcomes may require greater health literacy to manage over a significant time period, it is understandable that lower health literacy tends to correlate with worse outcomes for these types of health problems. These results generally align with the literature in other surgical fields and underscore the benefit of investing in health literacy supports to improve outcomes, especially for more under-resourced groups who may lack access to traditional health literacy materials.
